# Low bone mass and changes in the osteocyte network in mice lacking autophagy in the osteoblast lineage

**DOI:** 10.1038/srep24262

**Published:** 2016-04-11

**Authors:** Marilina Piemontese, Melda Onal, Jinhu Xiong, Li Han, Jeff D. Thostenson, Maria Almeida, Charles A. O’Brien

**Affiliations:** 1 Center for Osteoporosis and Metabolic Bone Diseases, University of Arkansas for Medical Sciences, Little Rock, Arkansas, United States of America; 2 Department of Biostatistics, University of Arkansas for Medical Sciences, Little Rock, Arkansas, United States of America; 3 Central Arkansas Veterans Healthcare System, Little Rock, Arkansas, United States of America

## Abstract

Autophagy maintains cell function and homeostasis by recycling intracellular components. This process is also required for morphological changes associated with maturation of some cell types. Osteoblasts are bone forming cells some of which become embedded in bone and differentiate into osteocytes. This transformation includes development of long cellular projections and a reduction in endoplasmic reticulum and mitochondria. We examined the role of autophagy in osteoblasts by deleting *Atg7* using an Osterix1-Cre transgene, which causes recombination in osteoblast progenitors and their descendants. Mice lacking *Atg7* in the entire osteoblast lineage had low bone mass and fractures associated with reduced numbers of osteoclasts and osteoblasts. Suppression of autophagy also reduced the amount of osteocyte cellular projections and led to retention of endoplasmic reticulum and mitochondria in osteocytes. These results demonstrate that autophagy in osteoblasts contributes to skeletal homeostasis and to the morphological changes associated with osteocyte formation.

The mammalian skeleton is continuously remodeled throughout life by the actions of osteoclasts, which resorb bone, and osteoblasts, which replace the bone matrix^1^. During the bone formation process, some of the osteoblasts are buried within the bone matrix and become osteocytes^2^. Osteocytes reside in lacunae and remain connected to one another, and with cells on the bone surface, via cellular projections that are contained within tubular cavities known as canaliculi. This network of osteocytes connected via their projections creates and maintains the lacunocanalicular network, which is thought to act as a mechanosensing system^3^. Recent studies have demonstrated that osteocytes also perform numerous non-mechanosensing functions including the control of bone remodeling and phosphate homeostasis^4^,^5^,^6^.

Macroautophagy, hereafter referred to as autophagy, is a stress-activated process that maintains cell function and homeostasis by recycling damaged organelles and macromolecules^7^. This process is thought to promote the health of long-lived cell types such as neurons and myocytes and a decline in autophagy in such cell types may contribute to the detrimental effects of aging^8^. Consistent with this, suppression of autophagy in neurons or myocytes mimics the effects of aging on the nervous system or muscle tissue, respectively^9^,^10^.

We have reported previously that genetic suppression of autophagy in osteocytes using a Dmp1-Cre transgene recapitulates many of the effects of aging on the skeleton, identifying autophagy as an important determinant of bone homeostasis and a decline in autophagy as a possible contributor to skeletal aging^11^. However, it remains unclear whether autophagy also plays a role in the differentiation or function of osteoblasts. Moreover, even though osteocyte formation was unaffected by deletion of Atg7 using the Dmp1-Cre transgene, it remains possible that loss of autophagy at a stage of osteoblast differentiation earlier than that targeted by Dmp1-Cre might affect the transition of osteoblasts to osteocytes and thereby proper formation of the osteocyte lacunocanalicular network.

To address these questions, we deleted the *Atg7* gene, which is essential for autophagy, using an Osterix 1 (Osx1)-Cre transgene, which targets cells at the earliest stages of commitment to the osteoblast lineage^12^. This maneuver caused a low bone mass phenotype that was more pronounced than that obtained using the Dmp1-Cre transgene and was associated with fractures. In addition, the lack of autophagy early in the osteoblast lineage greatly reduced the extent of osteocyte cellular projections and altered osteocyte cell body morphology, suggesting that autophagy is involved in the transition of osteoblasts into osteocytes.

## Methods

### Animal studies

Mice lacking autophagy in the osteoblast lineage were generated by crossing mice hemizygous for an Osx1-Cre transgene^12^ with mice heterozygous for an *Atg7*-flox allele^13^ to generate heterozygous *Atg7*-flox offspring with and without the Osx1-Cre allele. These offspring were then intercrossed to generate the following experimental mice: wild type (wt) mice, mice hemizygous for the Osx1-Cre allele, mice homozygous for the *Atg7*-flox allele, hereafter referred to as *Atg7*-f/f, and *Atg7*-f/f mice that were also hemizygous for the Osx1-Cre allele, hereafter referred to as Atg7^ΔOb^. To examine the role of oxidative stress in the skeletal phenotype, Atg7^ΔOb^ mice were also crossed with mice harboring a conditionally-activated transgene expressing mitochondria-targeted catalase (mCAT), an antioxidant enzyme^14^,^15^. Thus, in the offspring of this cross the Osx1-Cre transgene simultaneously deleted *Atg7* and activated the catalase transgene in the same cell populations (osteoblasts and osteocytes).

Offspring were genotyped by PCR using the following primer sequences: Cre-for, 5′-GCGGTCTGGCAGTAAAAACTATC-3′, Cre-rev, 5′-GTGAAACAGCATTGCTGTCACTT-3′, product size 102 bp; Hind-Fw, 5′-TGGCTGCTACTTCTGCAATGATGT-3′, Atg7-ex14-F, 5′-TCTCCCAAGACAAGACAGGGTGAA-3′, Pst-Rv, 5′-CAGGACAGAGACCATCAGCTCCAC-3′, product size 216 bp (WT) and 500 bp (floxed allele); mCAT-for, 5′-CTGAGGATCCTGTTAAACAATGC-3′ and mCAT-rev 5′-CTATCTGTTCAACCTCAGCAAAG-3′, product size 1,087 bp. All mice used in this study were in the C57BL/6J genetic background.

All animal studies were carried out in accordance with the policies of, and with approval from, the Institutional Animal Care and Use Committee of the University of Arkansas for Medical Sciences.

### Oxidative stress and autophagy flux assays

Intracellular reactive oxygen species (ROS) levels were measured in freshly isolated bone marrow cells from tibias using dichlorodihydrofluorescein diacetate dye^16^. The phophorylated form of p66^shc^ protein was quantified by immunoblot using protein isolated from L6 vertebrae^17^. Autophagic flux was measured using protein isolated from humeral cortical bone as follows. After removing the ends with a scalpel, bone marrow was flushed using a syringe filled with PBS, after which surface cells were removed by scraping with a scalpel. Cortical bone fragments were then frozen in liquid nitrogen and pulverized. The bone powder was then extracted by incubation in SDS-PAGE sample buffer (50 mM Tris-HCl, pH 6.8, 2% SDS, 6% glycerol, 1% β-mercaptolethanol, and 0.004% bromophenol blue) for 10 min at 100 °C. Proteins were resolved in SDS-polyacrylamide gels and electroblotted onto polyvinylidene difluoride membranes. Membranes were subsequently blocked with 5% non-fat dry milk in TBS with 0.1% Tween-20 and then incubated with primary antibodies and secondary antibodies. The following antibodies were used: anti-LC3A/B at 1:1000 (#4108, Cell Signaling), and anti-β-actin antibody at 1:5000 (# A5316, Sigma). Blots were developed using enhanced chemiluminescence and the intensity of the bands was quantified using a ChemiDoc XRS-plus system (Bio-Rad).

### Skeletal analysis and histomorphometry

Bone mineral density (BMD) was measured in live mice by dual-energy x-ray absorptiometry with a PIXImus Mouse Densitometer (GE Lunar Corp., Madison, WI) using the manufacturer’s software as described previously^18^. Growth curves were obtained by sequential BMD measurement of the same animals from 8 weeks to 24 weeks of age. Micro-CT analysis of cortical and trabecular architecture was performed in femurs and fourth lumbar vertebrae (L4), as previously described^19^. Biomechanical properties of femurs and L4 vertebrae were measured by 3-point bending and compression testing respectively, as previously described^20^. L1–L3 lumbar vertebrae were fixed, embedded undecalcified in methylmethacrylate, and histomorphometric examination was done on longitudinal sections with a digitizer tablet (OsteoMetrics, Inc., Decatur, GA) interfaced to a Zeiss Axioscope (Carl Zeiss, Thornwood, NY) with a drawing tube attachment, as previously described^21^. Terminology recommended by the Histomorphometry Nomenclature Committee of the American Society for Bone and Mineral Research was used in this study^22^.

### *In vitro* cell culture

Bone marrow cells were harvested from long bones and used to measure osteoblast differentiation of bone marrow precursors. 5 × 10^6^ bone marrow cells per well were plated in 12-well plates in α-MEM containing 15% fetal bovine serum, 1% penicillin/streptomycin/glutamine, 1% ascorbic acid, and 10 mM β-glycerolphosphate. Half of the culture medium was changed every 3 days. After 21 days, the cultures were fixed with phosphate buffered 10% formalin and then stained with an aqueous solution of 40 mM alizarin red. Messenger RNA and protein lysates were produced from parallel cultures lacking β-glycerolphosphate and harvested after 15 days, to evaluate respectively, osteoblast specific gene expression and LC3 conversion by western blot as previously described^18^. Catalase activity was also measured in primary bone marrow cells cultured under osteogenic conditions using the OxiSelect Catalase Activity Assay Kit, following the manufacturer’s instructions (Cell Biolabs). The ability of bone marrow cells to support osteoclast differentiation was evaluated by plating bone marrow cells as described above and adding vehicle or 10^−7^ M parathyroid hormone (PTH) for 12 days followed by RNA extraction and quantification of osteoclast-specific genes.

### RNA and genomic DNA isolation and TaqMan assay

Total RNA was prepared from L5 vertebrae by homogenization in 1.5 ml of Trizol Reagent (Life Technologies), followed by centrifugation and isopropanol precipitation according manufacturer’s instructions. Quantitation and the 260/280 ratio of the extracted RNA were determined using a Nanodrop instrument (Thermo Scientific). 500 ng of RNA was then used to synthesize first-strand cDNA using the High-Capacity cDNA Reverse Transcription Kit (Life Technologies) according to manufacturer’s directions. For detection of the mCAT transgene mRNA, we used a DNA-free-RNA isolation kit following manufacturer’s instructions (RNA Clean & Concentrator™-5, Zymo Research). cDNA was amplified by real-time PCR using TaqMan Universal PCR Master Mix (Life Technologies) as described previously^23^. The following TaqMan primer-probe sets were used: RANKL (Mm00441908_m1); Sost (Mm00470479_m1); osteoprotegerin (Mm00435452_m1); Dmp1 (Mm01208363_m1); Mepe (Mm02525159_s1); collagen 1a1 (Mm00801666_g1); osteocalacin (forward, 5′-GCTGCGCTCTGTCTCTCTGA-3′, reverse, 5′-TGCTTGGACATGAAGGCTTTG-3′, probe, 5′- FAM-AAGCCCAGCGGCC-NFQ-3′); osterix-1 (Mm00504574_m1); Runx2 (Mm00501584_m1); tartrate resistant acid phosphatase (Mm00475698_m1); calcitonin receptor (Mm00432271_m1); Cathepsin K (m00484036_m1); catalase (Hs00156308_m1) and the house-keeping gene ribosomal protein S2 (forward, 5′-CCCAGGATGGCGACGAT-3′, reverse, 5′-CCGAATGCTGTAATGGCGTAT-3′, probe, 5′-FAM-TCCAGAGCAGGATCC-NFQ-3′. Relative mRNA levels were calculated using the ΔCt method.

For genomic DNA isolation, bone pieces were decalcified in 14% EDTA for 1 week. Decalcified osteocyte-enriched bone or soft tissues were digested with proteinase K (0.5 mg/ml in 10 mM Tris, pH 8.0, 100 mM NaCl, 20 mM EDTA, and 1% SDS) at 55 °C overnight. Genomic DNA was then isolated by phenol/chloroform extraction and ethanol precipitation. To quantify *Atg7* gene deletion, the following custom Taqman assay for exon 14 was used: forward, 5′- ACCAGCAGTGCACAGTGA-3′, reverse, 5′- GCTGCAGGACAGAGACCAT-3′, probe, 5′-FAM-CTGGCCGTGATTGCAG-NFQ-3′. The custom *Atg7* assay was used in combination with a Taqman copy number reference assay, Tfrc (catalog number 4458367). All Taqman assays used in this study were obtained from Life Technologies.

### Osteocyte network analysis and immunofluorescence

Phalloidin staining of actin was performed on tibia. First, tibia were fixed in buffered 10% formalin for 24 hours, decalcified in 14% EDTA pH 7.1 for one week, stored in 30% sucrose solution, and then embedded in Cryo-Gel (Electron Microscopy Sciences, Hatfield, PA) for frozen sectioning. 20 μm thick frozen sections were cut and rinsed 3 times in PBS for 10 minutes. Sections were incubated in 0.2% Trition-X100 for 20 minutes with agitation, followed by washing in PBS. Sections were then incubated in 2% BSA for 30 minutes and incubated with Alexafluor 488-phalloidin (Molecular Probes) in 0.5% BSA for 48 hours at 4˚C. At the end of the incubation, the sections were rinsed with PBS and cover-slipped with Vectashield mounting medium containing DAPI (VectorLaboratories).

Fluorescein isothiocyanate (FITC) staining of the lacunocanalicular network was performed on femurs as previously described^24^. Briefly, femurs were harvested, placed in 10% phosphate buffered formalin solution for 24 hours, and then dehydrated in a series of ascending graded ethanol solutions. After removal of the distal end to allow efficient penetration of the staining solution, femurs were stained en block for 4 hours in 1% filtered FITC solution (fluorescein isothiocyanate isomer I, Sigma) with gentle agitation. Bones were then washed for 30 minutes in 100% ethanol to remove excess FITC solution and rinsed three times in methyl salicylate for 30, 45, and 60 minutes. After a quick rinse in xylene, femurs were embedded in methyl methacrylate and cross sections of 100 μm were obtained using a diamond saw, mounted and coverslipped using Eukitt mounting medium (Sigma).

Images of phalloidin- or FITC-stained bone sections were acquired using a Zeiss LSM 510 Meta/AxioVert 200 confocal microscope using a 40 × or 63 × oil objective and z-stacks were obtained using ZEN 2009 software. Measurements of osteocyte dendrites, osteocyte cell body, nuclear diameter, and osteocyte lacuna diameter were performed on flattened z-stacks (19 μm for tibia and 10 μm for femur) using ImageJ software. To quantify osteocyte dendrites, first a region of interest (ROI) containing only cortical bone was drawn. Then osteocyte cell bodies were excluded from the ROI. Finally, the total dendrite fluorescence inside the ROI was calculated by subtracting the background fluorescence in a region lacking osteocytes or dendrites from the integrated intensity of the entire region. This value was then divided by the area of the region of ROI. Three fields of cortical bone from each sample were quantified and 3–4 samples per genotype were used.

For endoplasmic reticulum staining, frozen tibial sections were permeabilized in PBS containing 0.4% Triton X-100 for 1 hour at RT with agitation and blocking was performed in 1% BSA/5% normal goat serum (Sigma) in PBS containing 0.4% Triton X-100 at 4 °C overnight. After rinsing in PBS, the sections were incubated with a primary antibody against KDEL (#12223, Abcam) diluted 1:250 in blocking buffer for 1 hour at RT. Sections were washed 3 times for 10 minutes with PBS containing 0.4% Triton X-100 and incubated with Alexa Fluor^®^ 594 AffiniPure Goat Anti-Mouse IgG (#115-585-003, Jackson Immunoresearch) diluted 1:1000 in blocking buffer for 1 hour at RT. Sections were rinsed in PBS and mounted in Vectashield with DAPI and imaged with confocal microscope using a 40× or 63× oil lens objectiveS. Z-stacks from 45 planes with 0.34 μm Z plane separation were obtained using ZEN 2009 software.

### Statistics

Data were analysed using two-way analysis of variance, one-way analysis of variance, nested analysis of variance, or Student’s t-test to detect statistically significant effects, after determining that the data were normally distributed and exhibited equivalent variances. In some cases, data were transformed to obtain normally-distributed data. Multiple comparisons were evaluated with Tukey or Holm-Sidak post hoc tests. *P*-values less than 0.05 were considered as significant. All values are reported as the mean ± S.D. Data that did not pass the normality test after transformation were evaluated using the Mann-Whitney Rank Sum Test.

## Results

### Deletion of *Atg7* early in the osteoblast lineage causes low bone mass

Mice harboring a conditional *Atg7* allele (*Atg7-f/f*) were crossed with Osx1-Cre transgenic mice^12^. The cell type-specificity of *Atg7* deletion was confirmed by demonstrating loss of *Atg7* genomic DNA in bone but not soft tissues (Supplementary Fig. S1). Functional suppression of autophagy can be demonstrated by reduced conversion of the unlipidated form of LC3 (I) to the lipidated form (II). Measurement of these proteins in either osteocyte-enriched bone or primary cultures of osteoblasts confirmed reduced conversion of LC3-I to LC3-II (Supplementary Fig. S1). Overall these results demonstrate suppression of autophagic flux in the osteoblasts and osteocytes of conditional knockout mice, designated Atg7^ΔOb^.

**Figure 1:**
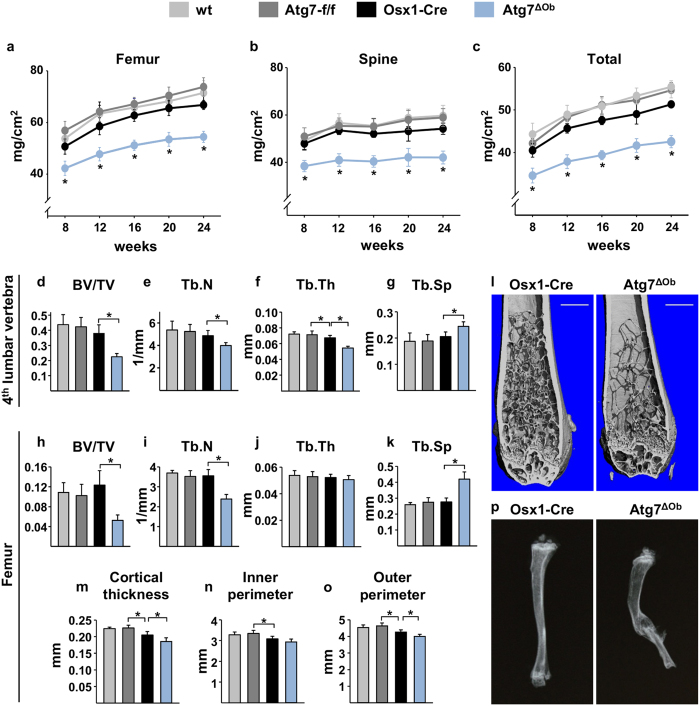
Deletion of *Atg7* in osteoblasts causes low bone mass and fractures. (**a–c**) BMD was measured monthly in the same cohort of mice by DXA beginning at 8 weeks of age until 24 weeks of age. Regions of interest were the right femur, the lumbar spine (T12-L6), and whole body excluding the head and neck. Female littermates of the following genotypes were used: wt (n = 7), *Atg7*-f/f (n = 16), Osx1-Cre (n = 8), and Atg7^ΔOb^ (n = 11). (**d–g**) μCT analysis of the cancellous bone of the 4^th^ lumbar vertebra of 6-month-old female littermates of the following genotypes: wt (n = 7), *Atg7*-f/f (n = 16), Osx1-Cre (n = 7), and Atg7^ΔOb^ (n = 11). BV/TV, bone volume per total tissue volume; Tb.N, trabecular number; Tb.Th, trabecular thickness; Tb.Sp, trabecular separation. (**h–k**) Cancellous bone measurements of distal femur of the same mice described in **d**. wt (n = 6), *Atg7*-f/f (n = 16), Osx1-Cre (n = 8), and Atg7^ΔOb^ (n = 10). (**l**) Representative μCT reconstructions of the distal femur. Scale bar = 1 mm. (**m–o**) Cortical bone measurements of mid-diaphyseal femur of the mice described in **d**. wt (n = 6), *Atg7*-f/f (n = 16), Osx1-Cre (n = 8), and Atg7^ΔOb^ (n = 10). (**p**) X-ray images of tibial bone showing a healed or healing fracture in the tibia of the Atg7^ΔOb^ mouse. Values are the mean ± sd. *P < 0.05 by one-way ANOVA.

Female Atg7^ΔOb^ mice and their control littermates, which included wild-type (wt), *Atg7*-f/f, or Osx1-Cre, were subjected to serial BMD measurements from 8 to 24 weeks of age and then euthanized to perform further skeletal analysis. Femoral, vertebral, and total BMD were low in Atg7^ΔOb^ mice compared to control groups at all time-points (Fig. 1a–c). There were no differences in body weight between Atg7^ΔOb^ and Osx1-Cre (Supplementary Fig. S2), which both weighed slightly less than wild-type controls, consistent with previously reported effects of the Osx1-Cre transgene on body weight^25^. Similar findings were observed in male mice (Supplementary Fig. S3).

MicroCT analysis revealed that cancellous bone volume was low in the spine and femur of female conditional knockout mice compared to controls (Fig. 1d–l). Femoral cortical thickness was also low compared with Osx1-Cre mice, and this was associated with a reduction in the outer cortical perimeter (Fig. 1m–o). These cortical bone changes were in addition to the effect of the Osx1-Cre transgene alone, which also caused low cortical thickness compared to *Atg7*-f/f littermates. In line with these results, biomechanical strength of femurs and isolated vertebrae was low, as revealed by 3-point bending and compression testing (Supplementary Fig. S4). Moreover, approximately half of the Atg7^ΔOb^ mice (11 of 23), but none of the control mice (0 of 12), displayed healed or healing fracture calluses in the tibia, indicative of fractures and a profound loss of strength in appendicular bones (Fig. 1p).

### Loss of autophagy suppresses bone remodeling

**Figure 2:**
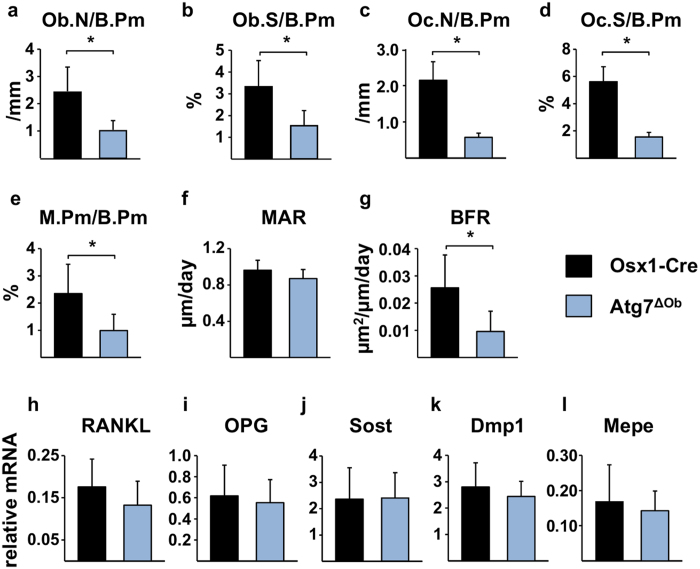
Deletion of *Atg7* in osteoblasts causes low bone remodeling. (**a–g**) Histomorphometric measurements were performed in thin sections of cancellous bone of the lumbar spine of 6-month-old female littermates of the following genotypes of Osx1-Cre (n = 4) and Atg7^ΔOb^ (n = 5) mice. Ob.N/B.Pm, osteoblast number per bone perimeter; Ob.S/B.Pm, osteoblast surface per bone perimeter; Oc.N/B.Pm, osteoclast number per bone perimeter; Oc.S/B.Pm, osteoclast surface per bone perimeter; M.Pm/B.Pm, mineralizing surface per bone perimeter; MAR, mineral appositional rate; BFR, bone formation rate. (**h–l**) Quantitative RT-PCR was used to measure the indicated mRNAs extracted from whole L5 vertebra of 6-month-old female littermates of the following genotypes of Osx1-Cre (n = 8) and Atg7^ΔOb^ (n = 11) mice. Values are the mean ± sd. *P < 0.05 by Student’s *t*-test.

Static and dynamic histomorphometry of cancellous bone of the spine revealed a low bone remodeling phenotype in Atg7^ΔOb^ mice, with reduced osteoblast and osteoclast number and surface (Fig. 2a–d). Quantification of tetracycline labeling showed reduced mineralized surface in the conditional knockout mice compared to the Osx1-Cre control, which resulted in a low bone formation rate (Fig. 2e–g). The differentiation capacity of osteoblast progenitors in the bone marrow was not different between conditional knockout mice and Osx1-Cre littermates as assessed by osteoblast-specific gene expression and mineralization (Supplementary Fig. S5). Similarly, osteoclast formation in bone marrow cultures was similar between these genotypes (Supplementary Fig. S5). Thus, the low osteoclast and osteoblast numbers *in vivo* do not appear to be due to cell intrinsic defects in the differentiation of either of these two cell types. Osteocytes produce a number of factors that control bone remodeling and mineralization^2^. There were no significant changes in the expression level of many of these factors, including RANKL, OPG, Sost, Dmp1 and Mepe, as measured in mRNA isolated from L5 vertebra (Fig. 2h–l).

### Expression of mitochondria-targeted catalase does not prevent low bone mass

Suppression of autophagy in other cells types often results in reduced recycling of mitochondria leading to accumulation of damaged mitochondria and oxidative stress. Consistent with this, impairment of autophagy in the entire osteoblast lineage was associated with elevated levels of reactive oxygen species (ROS) in the bone marrow and increased phosphorylation of the redox sensitive protein p66^shc^ measured in bone protein lysate (Supplementary Fig. S6). To determine whether this increase in oxidative stress contributes to the low bone mass caused by *Atg7* deletion, Atg7^ΔOb^ mice were crossed with mice harboring a conditionally-activated transgene expressing mitochondrial-targeted catalase (mCAT), an antioxidant enzyme^15^. Thus, in the offspring of this cross, the Osx1-Cre transgene simultaneously deletes *Atg7* and activates the catalase transgene in the same cell populations (osteoblasts and osteocytes).

**Figure 3:**
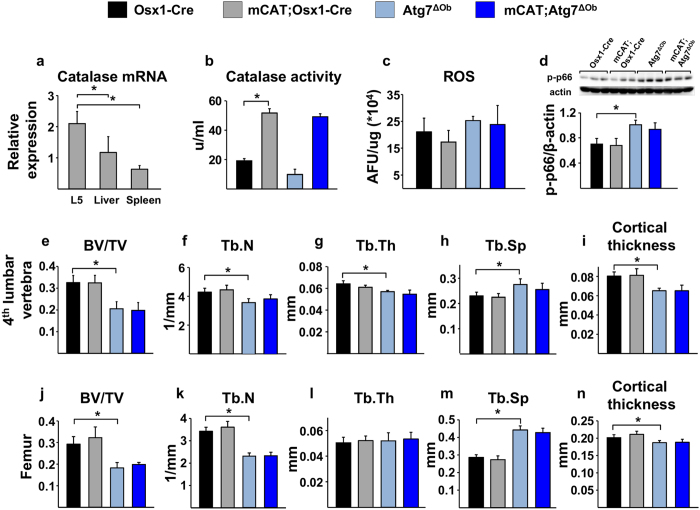
Mitochondrial-targeted catalase does not alter the impact of autophagy suppression. (**a**) Catalase mRNA was quantified by RT-PCR in the indicated tissues of mCAT;Osx1-Cre mice (n = 7). (**b**) Catalase activity measured in bone marrow-derived osteoblasts of Osx1-Cre (n = 3), mCAT;Osx1-Cre (n = 3), Atg7^ΔOb^ (n = 3), and mCAT;Atg7^ΔOb^ (n = 3) mice. (**c**) ROS measured in bone marrow cells of Osx1-Cre (n = 3), mCAT;Osx1-Cre (n = 4), Atg7^ΔOb^ (n = 3), and mCAT;Atg7^ΔOb^ (n = 4) mice. (**d**) Phosphorylated p66^shc^ measured by immunoblot of protein extracted from whole L6 vertebra of Osx1-Cre (n = 3), mCAT;Osx1-Cre (n = 3), Atg7^ΔOb^ (n = 3), and mCAT;Atg7^ΔOb^ (n = 3) mice. (**e–i**) μCT measurements of L4 vertebra from Osx1-Cre (n = 6), mCAT;Osx1-Cre (n = 6), Atg7^ΔOb^ (n = 6), and mCAT;Atg7^ΔOb^ (n = 6) mice. BV/TV, bone volume per total tissue volume; Tb.N, trabecular number; Tb.Th, trabecular thickness; Tb.Sp, trabecular separation. (**j–n**) μCT measurements of femurs from the same mice described in **e**, Osx1-Cre (n = 6), mCAT;Osx1-Cre (n = 6), Atg7^ΔOb^ (n = 6), and mCAT;Atg7^ΔOb^ (n = 6) mice. All measurements were performed in 6-month-old female littermates. Values are the mean ± sd. *P < 0.05 by one-way ANOVA.

The catalase transgene was highly expressed in the bone of mCAT;Atg7^ΔOb^ mice and catalase activity, measured in bone marrow stromal cells cultured in osteogenic medium, was greatly elevated compared to control littermates, confirming activation of the transgene (Fig. 3a,b). In this experiment, ROS levels measured in the bone marrow were not different between genotypes but phosphorylation of p66shc protein was increased in Atg7^ΔOb^ mice (Fig. 3c,d). However, this increase was not affected by mCAT expression (Fig. 3d). Thus, mitochondrial expression of catalase in osteoblasts and osteocytes was not sufficient to block the increase in oxidative stress, measured in whole bone, caused by suppression of autophagy.

Serial BMD analysis up to 24 weeks of age confirmed low bone mass in the femur and spine of Atg7^ΔOb^ mice compared to Osx1-Cre control littermates (Supplementary Fig. S7). Expression of mCAT in Atg7^ΔOb^ mice did not alter the low bone mass caused by suppression of autophagy (Supplementary Fig. S7). Analysis of vertebral and femoral architecture confirmed low trabecular bone volume and cortical thickness in Atg7^ΔOb^ mice, and expression of mCAT did not prevent any of these changes (Fig. 3e–n). Consistent with this, the prevalence of fractures in tibias of conditional knockout mice was similar in conditional knockout mice expressing catalase (Supplementary Table S1). Together these results suggest that autophagy exerts its positive effects on the skeleton by mechanisms other than suppression of H_2_O_2_ levels in the mitochondria of osteoblasts and osteocytes.

### Suppression of autophagy alters morphology of the osteocyte network

Autophagy plays a key role in the differentiation process of several cell types including erythrocytes, adipocytes, and lymphocytes^26^,^27^,^28^,^29^,^30^. Moreover, autophagy in erythrocyte progenitors contributes to the removal of cytoplasmic organelles, such as mitochondria, that is associated with maturation of this cell type^26^,^31^,^32^. A similar reduction in cytoplasmic organelles occurs as osteocytes differentiate from osteoblasts^33^. Therefore, we measured different features of osteocyte biology to determine whether loss of autophagy in osteoblasts altered osteocyte formation or function.

**Figure 4:**
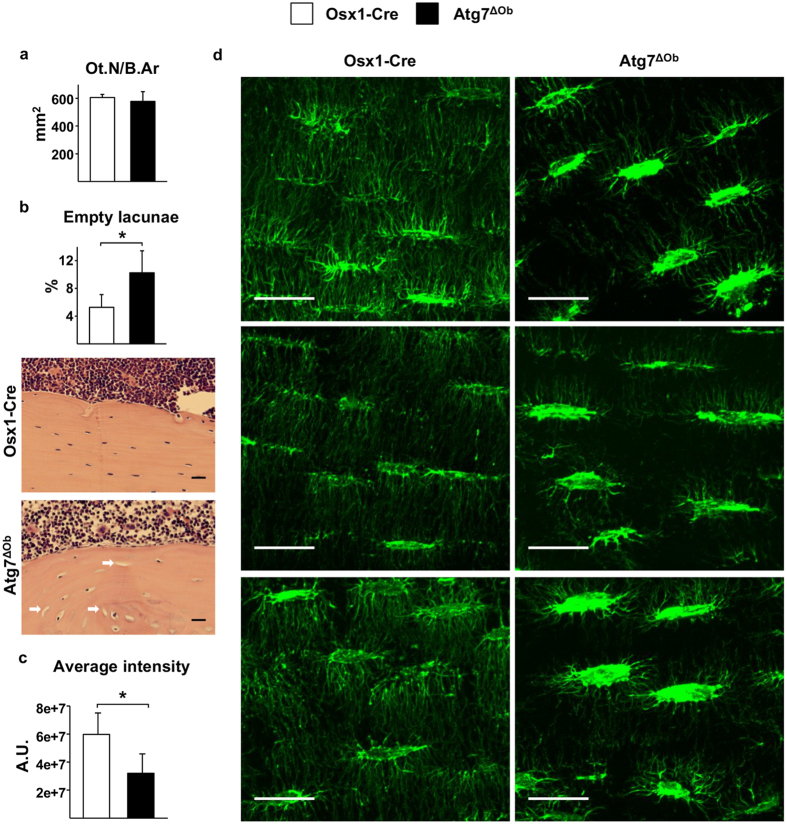
Loss of autophagy in osteoblast-lineage cells disrupts the osteocyte network. (**a**) Histomorphometric measurement of osteocyte density (Ot.N/B.Ar, osteocyte number per bone area) in tibial cortical bone of Osx1-Cre (n = 4) and Atg7^ΔOb^ (n = 5) mice. (**b**) Histomorphometric measurement and representative images of empty osteocyte lacunae in tibial cortical bone of same mice described in **a**. Values are the mean ± sd. *P < 0.05 by Student’s *t*-test. (**c**) Average intensity of osteocyte projections in tibial cortical bone sections from Osx1-Cre (n = 4) and Atg7^ΔOb^ (n = 4) mice stained with phallodin-Alexa488. Values are the mean ± sd. *P < 0.05 by Nested analysis. (**d**) Representative images of sections used to obtain values shown in **c**. Each image is from a different mouse. Size bar = 20 μm. All measurements were performed in 6-month-old female littermates.

The density of osteocytes per bone area was not different between Atg7^ΔOb^ mice and Osx1-Cre littermates (Fig. 4a). However, the number of empty lacunae, which is an indicator of osteocyte death^34^, was higher in the bones of conditional knockout mice (Fig. 4b). Next we examined the density of osteocyte cell projections, which are formed during the process of embedding into bone matrix, by staining with fluorescently-tagged phalloidin^35^. The average intensity of osteocyte projections normalized to bone area, which likely reflects the number and size of osteocyte cellular projections, was significantly lower in the conditional knockout mice compared with controls (Fig. 4c,d). These results suggest that formation or maintenance of the osteocyte network was disrupted by deletion of *Atg7* in osteoblasts.

We noted previously that deletion of *Atg7* using a Dmp1-Cre transgene did not alter osteocyte maturation or formation of the osteocyte network^11^,^18^. However, the latter observation was limited to qualitative evaluation of phalloidin-stained images. Therefore, we quantified the density of osteocyte projections in Dmp1-Cre;*Atg7*-f/f mice and their littermate controls using the same approach as we used in the current study. Although there was a trend towards reduced area, this difference was not significant (Supplementary Fig. S8). Thus, loss of autophagy in osteoblasts, but not in osteocytes, altered osteocyte projections.

**Figure 5:**
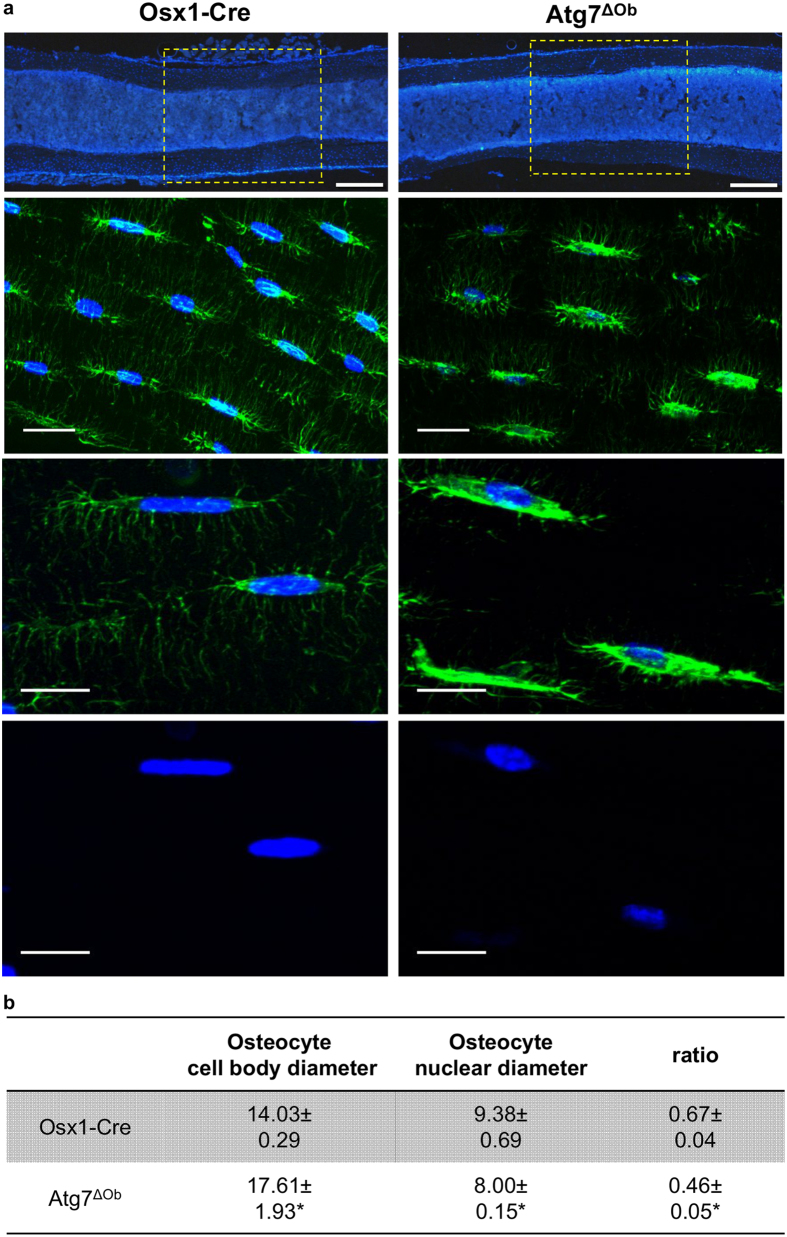
Loss of autophagy in osteoblasts alters the nuclear to cytoplasmic ratio in osteocytes. (**a**) Representative images of thick sections of tibial cortical bone from Osx1-Cre and Atg7^ΔOb^ littermates stained with phalloidin-Alexa488 and DAPI. Top panels show a region of interest from tibia diaphysis chosen for the subsequent osteocyte analysis. Size bar = 200 μm. Second and third panels are from different mice and the lower panel shows the DAPI signal only from the same samples as the third panels. Size bar = 20 μm. (**b**) Table of measurements of osteocyte cell body diameter, nuclear diameter, and the ratio between the two in tibial cortical bone from Osx1-Cre (n = 4) and Atg7^ΔOb^ (n = 4) littermates. All mice were 6-month-old females. Values are the mean ± sd. *P < 0.05 by Nested analysis.

**Figure 6:**
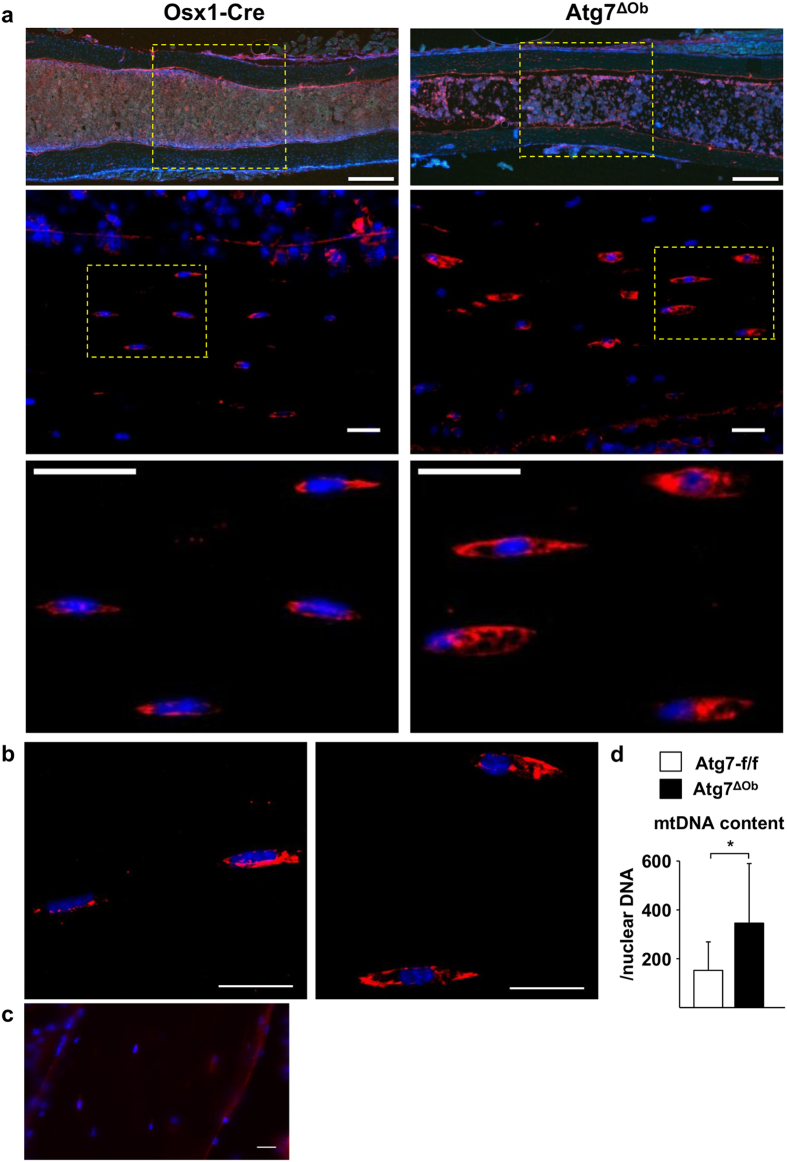
Suppression of osteoblast autophagy inhibits osteocyte maturation. (**a**) Representative images of thick sections of tibial cortical bone from Osx1-Cre and Atg7^ΔOb^ littermates stained with anti-KDEL antibody and DAPI. Top panels show a region of interest from tibia diaphysis chosen for the subsequent osteocyte analysis. Size bar = 200 μm. The third panel shows an enlargement of the area surrounded by the yellow-dotted box in the second panel. Size bar = 20 μm. (**b**) Images from a second mouse for each genotype. Size bar = 20 μm. (**c**) Negative control staining of cortical bone section omitting the anti-KDEL antibody. Size bar = 20 μm. (**d**) Quantitative PCR analysis of mitochondrial DNA (mtDNA), normalized to nuclear DNA, from osteocyte-enriched cortical bone from Atg7-f/f (n = 9) and Atg7^ΔOb^ (n = 9) littermates. All measurements were performed in 6-month-old littermates. Values are the mean ± sd. *P < 0.05 by Student’s *t*-test.

We also noted that the shape of osteocyte nuclei appeared more round and off-centre in conditional knockout mice compared with controls (Fig. 5a). Quantification of this phenomenon revealed that osteocyte cell bodies were larger, but nuclei were smaller, in conditional knockout mice compared to controls (Fig. 5b). One possible cause for this phenomenon could be delay or prevention of the reduction of cytoplasmic components normally associated with osteocyte maturation. Consistent with this idea, the extent of endoplasmic reticulum (ER), as measured by anti-KDEL staining, was greatly expanded in Atg7^ΔOb^ mice (Fig. 6a–c). Similarly the amount of mitochondrial DNA present in osteocyte-enriched bone preparations was higher in conditional knockout mice compared with controls (Fig. 6d). Together this evidence suggests that the reduction in cytoplasmic components that normally occurs during osteocyte formation was delayed or prevented by loss of autophagy in osteoblasts.

Phalloidin staining labels actin and can thus detect changes only in cellular structures within the lacunocanalicular network. To directly visualize the lacunocanalicular network itself, we stained bones from conditional knockout mice and controls with FITC^24^. Using this approach we confirmed the increase in the size of osteocyte lacunae in Atg7^ΔOb^ mice (Supplementary Fig. S9). However, there was no difference in the appearance or staining intensity of the canaliculi in Atg7^ΔOb^ mice compared to Osx1-Cre controls (Supplementary Fig. S9). This latter finding suggests that it is the osteocyte cellular projections themselves, but not canalicular structure, that is altered by suppression of autophagy in osteoblasts.

## Discussion

We have shown previously that suppression of autophagy in osteocytes causes low bone mass and that this is associated with low bone remodeling^11^,^18^. However, the transformation of osteoblasts into osteocytes was not obviously affected by this maneuver. Here we demonstrate that suppression of autophagy early in the osteoblast lineage causes an even greater reduction in bone mass. The reduction in bone mass and strength was so severe that approximately fifty percent of the conditional knockout mice experienced fractures. Coincidently, suppression of autophagy early in the osteoblast lineage also altered the structure of the osteocyte network. Previous studies have suggested that such disruptions can compromise bone strength independently of changes in bone mass and architecture^21^. Therefore the extremely low bone strength of Atg7^ΔOb^ mice may result from the combination of low bone mass and the changes in the osteocyte network.

The Osx1-Cre transgenic mouse line used in these studies exhibits a basal skeletal phenotype^25^,^36^,^37^. For example, these mice are smaller than non-transgenic littermates and have reduced cortical thickness^25^. In addition, they display delayed mineralization of craniofacial bones shortly after birth but this phenotype appears to resolve as the animals mature^37^. More important to the studies described here, a recent analysis revealed fracture calluses in the scapula, ribs, and fibula in approximately 80% of 6 day old Osx1-Cre mice^36^. In the current study, we did not observe any tibial fractures in adult Osx1-Cre littermates. Nonetheless, it remains possible that the susceptibility of Atg7^ΔOb^ mice to tibial fractures is due in part to an additive effect of the Osx1-Cre basal skeletal phenotype and that caused by suppression of autophagy in osteoblast-lineage cells.

Two major morphological differences were observed between osteocytes in control versus conditional knockout mice. First, the density of osteocyte projections was reduced in cortical bone of Atg7^ΔOb^ mice. Second, osteocyte cell body diameter was elevated in these mice and this was associated with reduced nuclear diameter. A plausible explanation for the latter changes is that reduced degradation of the ER and other cytoplasmic components such as mitochondria, which normally occurs as osteocytes mature, did not occur efficiently in the conditional knockout mice^33^. This idea is supported by the increased amount of mitochondrial DNA and ER in osteocytes from these animals. Moreover, recent studies have demonstrated a critical role for autophagy in ER turnover and that suppression of autophagy results in ER expansion in several cell types, including mouse embryonic fibroblasts, osteosarcoma cells, and lymphocytes^38^,^39^. The distorted nuclear morphology may be an indirect consequence of retention of the cytoplasmic components. Together these results suggest that the reduction in cytoplasmic components that occurs during osteocyte maturation depends on autophagy. This requirement may be analogous to the requirement of autophagy to remove cytoplasmic organelles during maturation of mammalian reticulocytes and erythrocytes^26^,^31^,^32^.

It is unclear why these changes were not observed when *Atg7* was deleted using the Dmp1-Cre transgene. The Dmp1-Cre transgene becomes active during the late stages of osteoblast differentiation^40^,^41^. Therefore, one possible explanation is that even after the *Atg7* gene was deleted in mature osteoblasts, sufficient Atg7 protein remained to allow autophagy to continue until osteocytes were fully formed. In other words, residual autophagy may have still been present for a limited time after Cre-mediated recombination. In contrast, deletion of *Atg7* at the earliest stages of osteoblast differentiation would almost certainly result in a complete loss of Atg7 protein before the beginning of the transition from osteoblast to osteocyte, a conclusion supported by the strong reduction in LC3 lipidation observed in our osteoblast cultures.

Our findings are consistent with the idea that maintenance of osteocyte projections is influenced by autophagy-driven metabolism. In wild-type mice, osteocyte projections form before removal of cytoplasmic components^42^. Therefore, formation of the projections must not depend on material supplied by autophagic digestion of cytoplasm products. In addition, if initial formation of the canalicular network depends on osteocyte projections, then the normal canalicular morphology in the conditional knock out mice suggests that at the time of canalicular formation, osteocyte projections were present in normal numbers in these mice. The reduction in osteocyte projections observed by phalloidin staining could be explained either by retraction or degradation of projections or by loss of actin filaments within such projections. *In vitro* studies suggest that actin filaments are required to maintain the number and structure of osteocyte projections^43^, arguing against the latter possibility.

The functional significance of the changes in osteocyte morphology is unclear but they were associated with profound reductions in bone remodeling and mass, as well as an increase in osteocyte death. We found no changes in the ability of progenitors to differentiate into osteoclasts or osteoblasts *in vitro*, suggesting that the low bone remodeling was not due to an intrinsic defect in either of these two cell types. Osteocytes have been shown to control osteoclast formation via production of RANKL, which is essential for osteoclast formation and a major determinant of osteoclast number^44^. In addition, osteocytes are also able to control osteoblast formation via production of the Wnt antagonist sclerostin^45^. However, mRNA levels of both of these genes were unaffected by deletion of *Atg7*. Nonetheless, it is possible that the change in osteocyte morphology, in and of itself, may have influenced bone remodeling. RANKL is produced as both membrane-bound and soluble protein and *in vitro* studies suggest that it is the membrane-bound form that drives osteoclast formation^46^,^47^. If this were the case *in vivo*, then osteocytes may present RANKL to osteoclast progenitors only on those projections that extend into the bone marrow cavity or those that have contact with blood vessels^2^. Since the extent of osteocyte projections is reduced in Atg7^ΔOb^ mice, then the amount of RANKL available to osteoclast progenitors may also be reduced leading to a low osteoclast number.

It is not clear how changes in osteocyte morphology might alter osteoblast formation or function. A previous study reported that suppression of autophagy in osteoblast-lineage cells suppresses osteoblast differentiation^48^. Specifically, deletion of FIP200 using the same Osx1-Cre transgenic line used in our study reduced bone mass as well as osteoblast differentiation as measured by mineralization of osteoblast cultures and osteoblast-specific gene expression^48^. FIP200 is a protein required for the initiation of autophagosome formation and is also involved in the control of several signalling pathways^49^. Deletion of Atg5, another gene essential for autophagy, using a Col1a1-Cre transgene resulted in low bone mass and osteoblastic cells from these mice displayed reduced mineralization in culture^50^. However, in the latter study, osteoblast differentiation, as measured by expression of osteoblast-specific genes, was actually elevated in the conditional knockout mice. Fractures were not observed in mice when either FIP200 or Atg5 genes were deleted from osteoblast-lineage cells. In the present study we observed no change in osteoblast-specific gene expression or mineralization in culture or in bone mineralization *in vivo*, suggesting that osteoblast differentiation and function were not affected. The reasons for the different outcomes are not clear, but in the case of FIP200 deletion the differences may be due to suppression of processes other than autophagy^49^.

Suppression of autophagy induces oxidative stress in many cell types and this is often associated with increased abundance of damaged mitochondria^51^,^52^,^53^. Similarly, we noted markers of increased oxidative stress in the bones of mice lacking *Atg7* in the entire osteoblast lineage or just in mature osteoblasts and osteocytes^11^. We have previously used the mCAT transgene to suppress H_2_O_2_ in osteoclast-lineage cells and this prevented the bone loss caused by estrogen deficiency^54^. Moreover, this transgene has been used successfully to suppress oxidative damage in a variety of mouse models^15^,^55^,^56^,^57^,^58^. Thus, in the present study it is likely that the mCAT transgene suppressed levels of H_2_O_2_ in the mitochondria of osteoblast-lineage cells. However, this was not sufficient to alter any of the skeletal effects caused by suppression of autophagy in these cells. Based on this, it appears that mitochondrial oxidative stress is not a contributing factor to the detrimental effects of autophagy suppression on the skeleton.

In summary, we have shown that suppression of autophagy early in the osteoblast lineage leads to a profound reduction in bone mass associated with reduced maturation of osteocyte morphology. Whether the altered morphology of osteocytes contributes to the reductions in bone remodeling or strength will require a more complete understanding of how osteocytes communicate with osteoclast and osteoblast progenitors. For example, whether osteocytes utilize the membrane-bound form of RANKL, its soluble form, or both, will need to be determined. It will also be important to determine whether the failure of osteocyte maturation leads to activation of pathways that impact cell survival or function.

## Additional Information

**How to cite this article**: Piemontese, M. *et al.* Low bone mass and changes in the osteocyte network in mice lacking autophagy in the osteoblast lineage. *Sci. Rep.* **6**, 24262; doi: 10.1038/srep24262 (2016).

## Supplementary Material

Supplementary Information
