# Supplementary material for: Low bone mass and changes in the osteocyte network in mice lacking autophagy in the osteoblast lineage
Source: Sci Rep. 2016 Apr 11;6:24262. doi: 10.1038/srep24262 (PMC4827128; doi:10.1038/srep24262)
Supplement: Supplementary Information [file srep24262-s1.pdf]

Supplementary information for: Low bone mass and changes in the osteocyte network in mice lacking autophagy in the osteoblast lineage

Marilina Piemontese, Melda Onal, Jinhu Xiong, Li Han, Jeff D. Thostenson, Maria Almeida, and Charles A. O’Brien

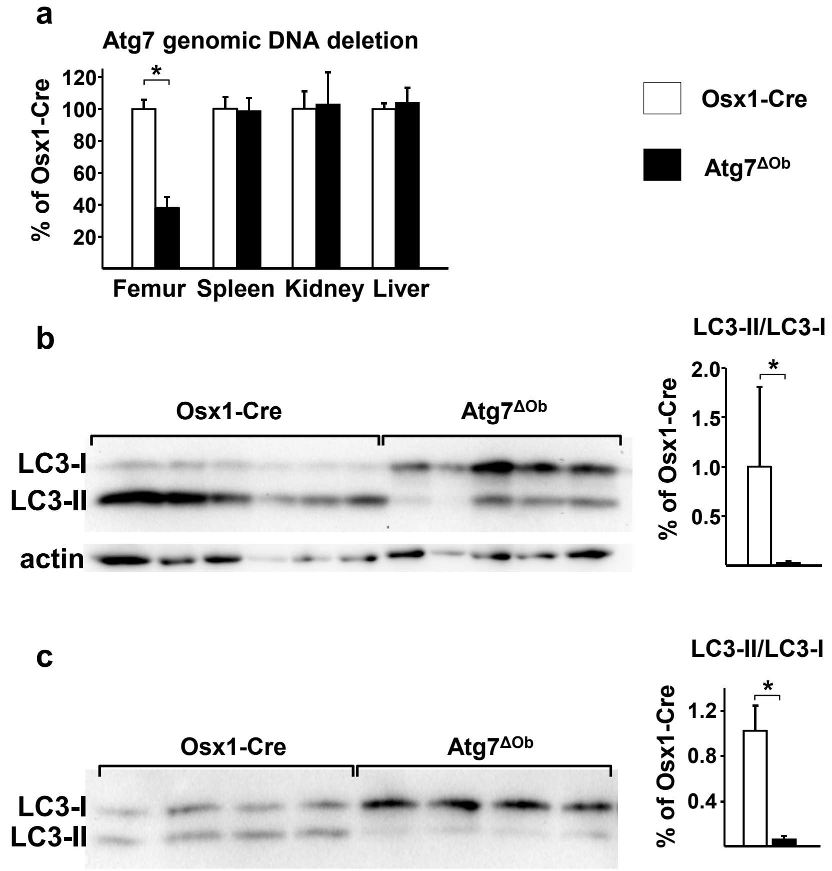

Figure S1. Confirmation that Osx1-Cre deletes Atg7 and suppresses autophagy in osteoblast-lineage cells. (**a**) Quantitative PCR of loxP-flanked Atg7 exon in genomic DNA from femoral cortical bone and the indicated soft tissues. Tissues were obtained from Osx1-Cre (n = 6) and $Atg7^{\Delta Ob}$ (n = 5) littermates. (**b**) Immunoblot of LC3 in protein extracted from humeral cortical bone of Osx1-Cre (n = 6) and $Atg7^{\Delta Ob}$ (n = 5) littermates. Each lane is from a single mouse. Quantification of the ratio of LC3-II to LC3-I band intensity is shown at the right. (**c**) Immunoblot of LC3 in protein extracted from primary bone marrow cultures of Osx1-Cre (n = 4) and $Atg7^{\Delta Ob}$ (n = 4) littermates. Each lane represents protein extracted from the culture on an individual mouse. Quantification of the ratio of LC3-II to LC3-I band intensity is shown at the right. All mice were 9-month-old male littermates. Values are the mean ± sd. **P* < 0.05 by Student's t-test.

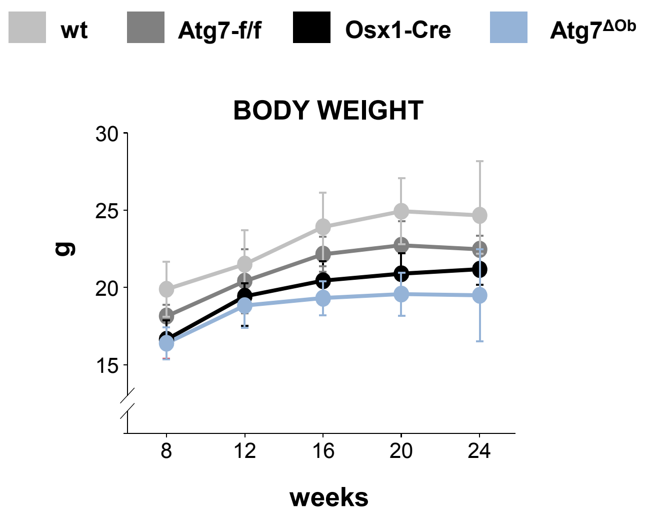

Figure S2. Female mice lacking Atg7 in osteoblasts have normal body weight. Body weight was measured monthly in the same cohort of mice beginning at 8 weeks of age until 24 weeks of age. Female littermates of the following genotypes were used: wt (n = 7), Atg7-f/f (n = 16), Osx1-Cre (n = 8), and Atg7$^{\Delta Ob}$ (n = 11). Values are the mean ± sd.

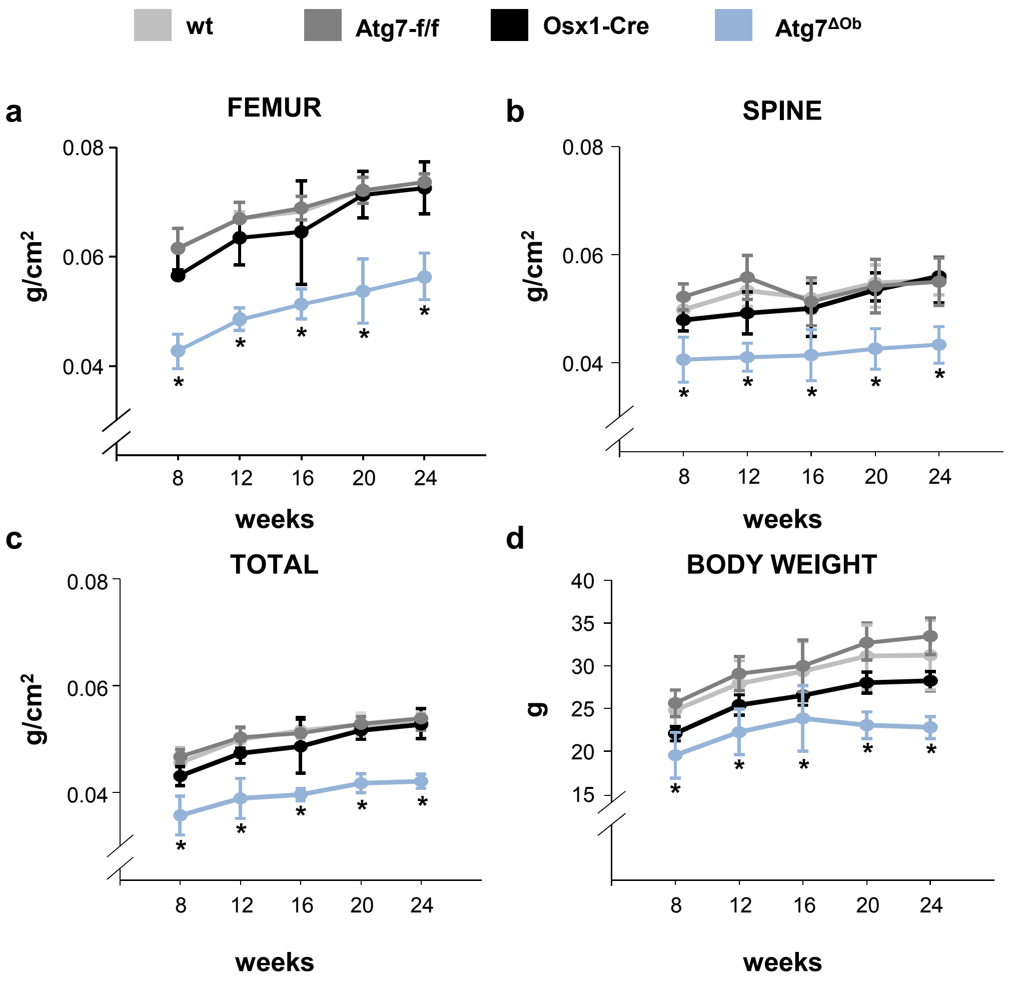

Figure S3. Male mice lacking Atg7 in osteoblasts have low bone mass. (**a-c**) BMD was measured monthly in the same cohort of mice by DXA beginning at 8 weeks of age until 24 weeks of age. Regions of interest were the right femur, the lumbar spine (T12-L6), and whole body excluding the head and neck. Male littermates of the following genotypes were used: wt (n = 9), Atg7-f/f (n = 13), Osx1-Cre (n = 5), and Atg7$^{\Delta Ob}$ (n = 12). (**d**) Body weight was measured in the same mice described in **a**. Values are the mean ± sd. **P* < 0.05 by two-way ANOVA at each time point.

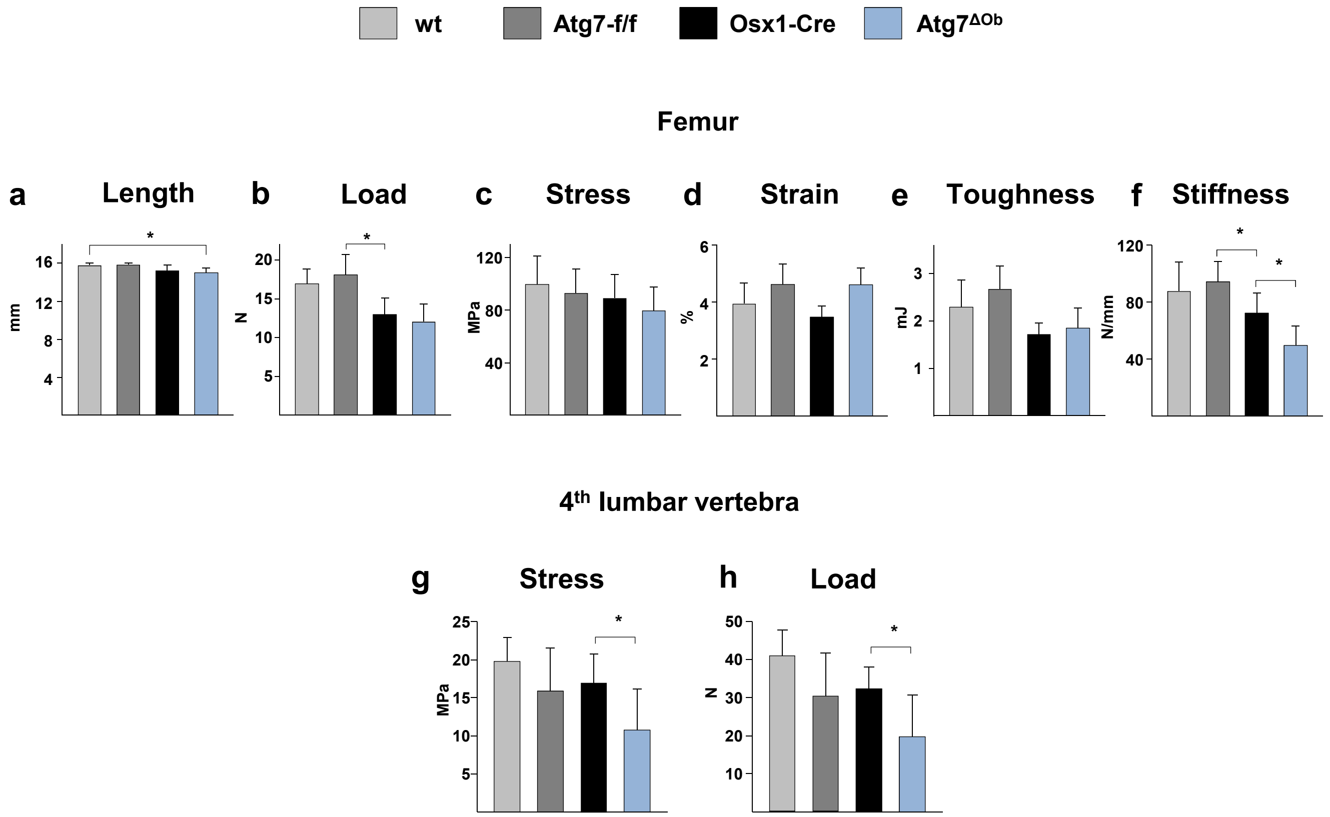

Figure S4. Loss of osteoblast autophagy lowers bone strength. (**a**) Femur length was measured in 6-month-old female mice of the following genotypes using a micrometer: wt (n = 6), Atg7-f/f (n = 13), Osx1-Cre (n = 5), and Atg7$^{\Delta Ob}$ (n = 9). (**b-f**) Femurs described in **a** were subjected to three-point bending using a material testing instrument to yeild the indicated measurements. (**g-h**) L4 vertebra from the mice described in **a** were subjected to compression testing using a material testing instrument to yeild the indicated measurements. wt (n = 7), Atg7-f/f (n = 14), Osx1-Cre (n = 7), and Atg7$^{\Delta Ob}$ (n = 10). Values are the mean ± sd. *$P < 0.05$ by one-way ANOVA.

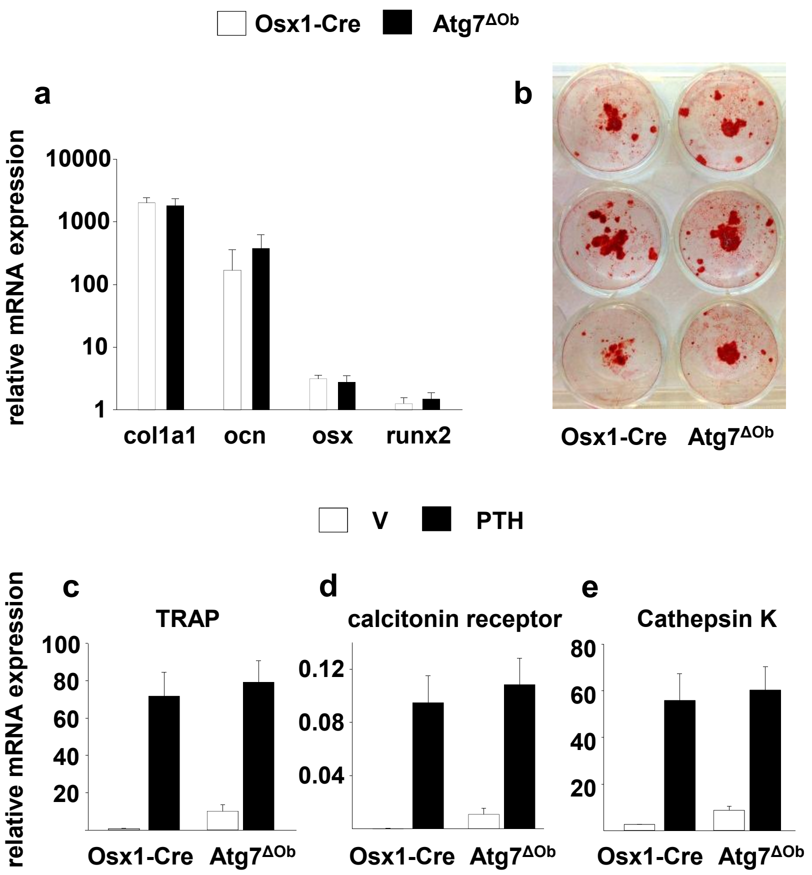

Figure S5. Loss of osteoblast autophagy does not alter osteoblast or osteoclast differentiation in vitro. (**a**) Quantitative RT-PCR of collagen 1a1 (col1a1), osteocalcin (ocn), osterix 1 (osx), and runx2 mRNA in primary bone marrow osteoblast cultures. Cultures for each genotype were performed in triplicate using bone marrow cells pooled from 3 mice of each genotype. *P < 0.05 using Student's t-test (**b**) Alizarin Red staining of primary bone marrow cells cultured for 21 days in osteoblast differentiation medium (n = 3 wells/genotype). (**c-e**) Quantitative RT-PCR of TRAP, calcitonin receptor, and cathepsin K mRNA in bone marrow cultures treated with vehicle or PTH for 11 days to induce osteoclast formation (n = 4 wells per group). *P < 0.05 using two-way ANOVA

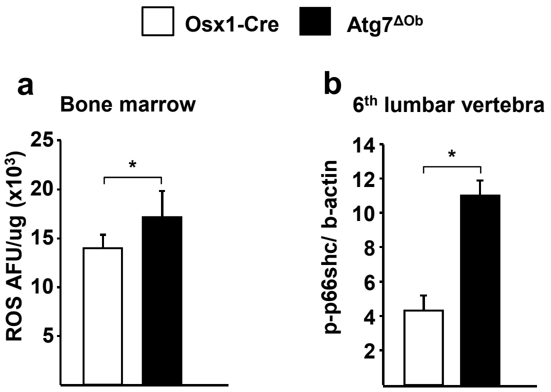

Figure S6. Loss of osteoblast autophagy caused oxidative stress. (**a**) ROS in bone marrow isolated from tibia of 6-month-old female Osx1-Cre (n = 5) and Atg7ΔOb (n = 6) mice. (**b**) Quantification of phospho-p66shc in immunoblot of protein from L6 vertebra 6-month-old female Osx1-Cre (n = 4) and Atg7ΔOb (n = 5) mice. *$P < 0.05$ using Student's *t*-test.

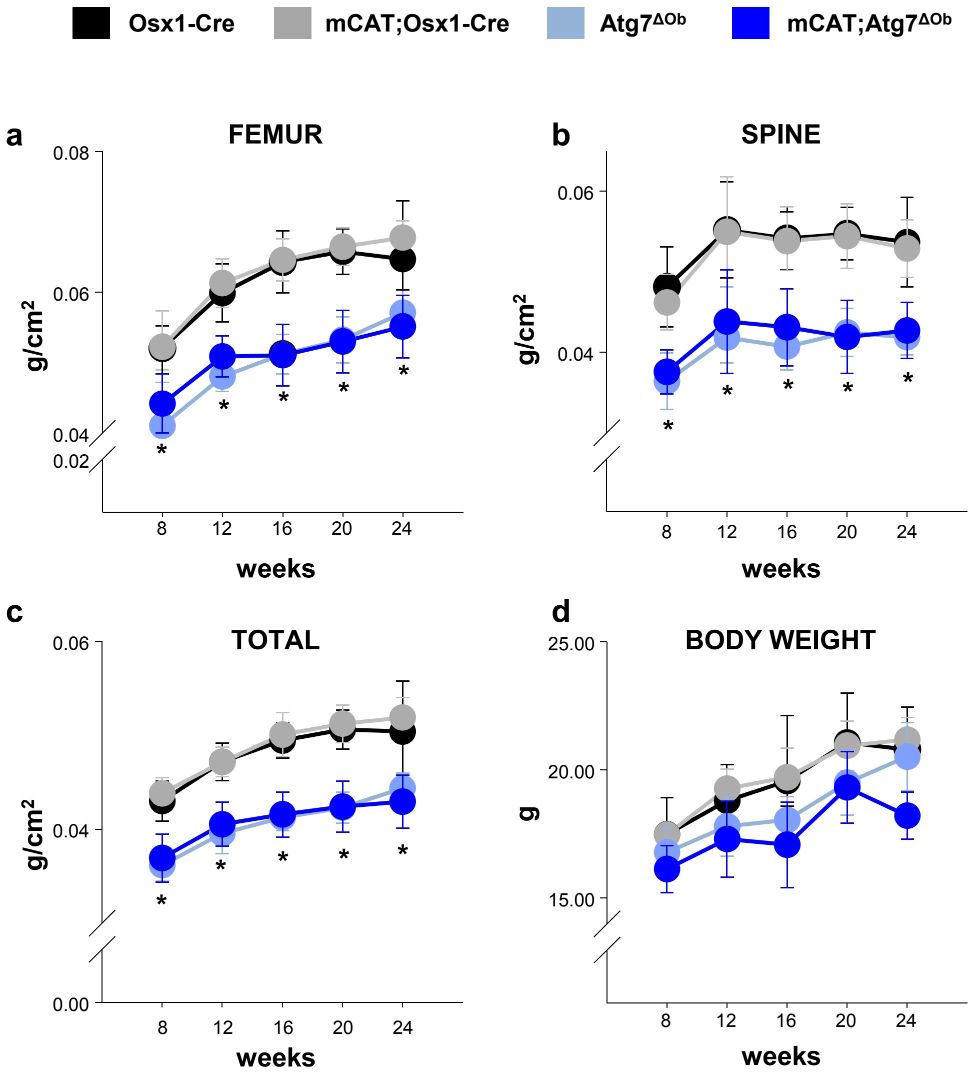

Figure S7. mCAT expression does not rescue low BMD of Atg7$^{\Delta Ob}$ mice. (**a-d**) BMD was measured monthly in the same cohort of mice by DXA beginning at 8 weeks of age until 24 weeks of age. Regions of interest were the right femur, the lumbar spine (T12-L6), and whole body excluding the head and neck. Female littermates of the following genotypes were used: : Osx1-Cre (n = 7), mCAT;Osx1-Cre (n = 6), Atg7$^{\Delta Ob}$ (n = 6), and mCAT; Atg7$^{\Delta Ob}$ (n = 6). (**b**) Body weight was measured in the same mice described in **a**. Values are the mean ± sd. *P < 0.05 by two-way ANOVA at each time point.

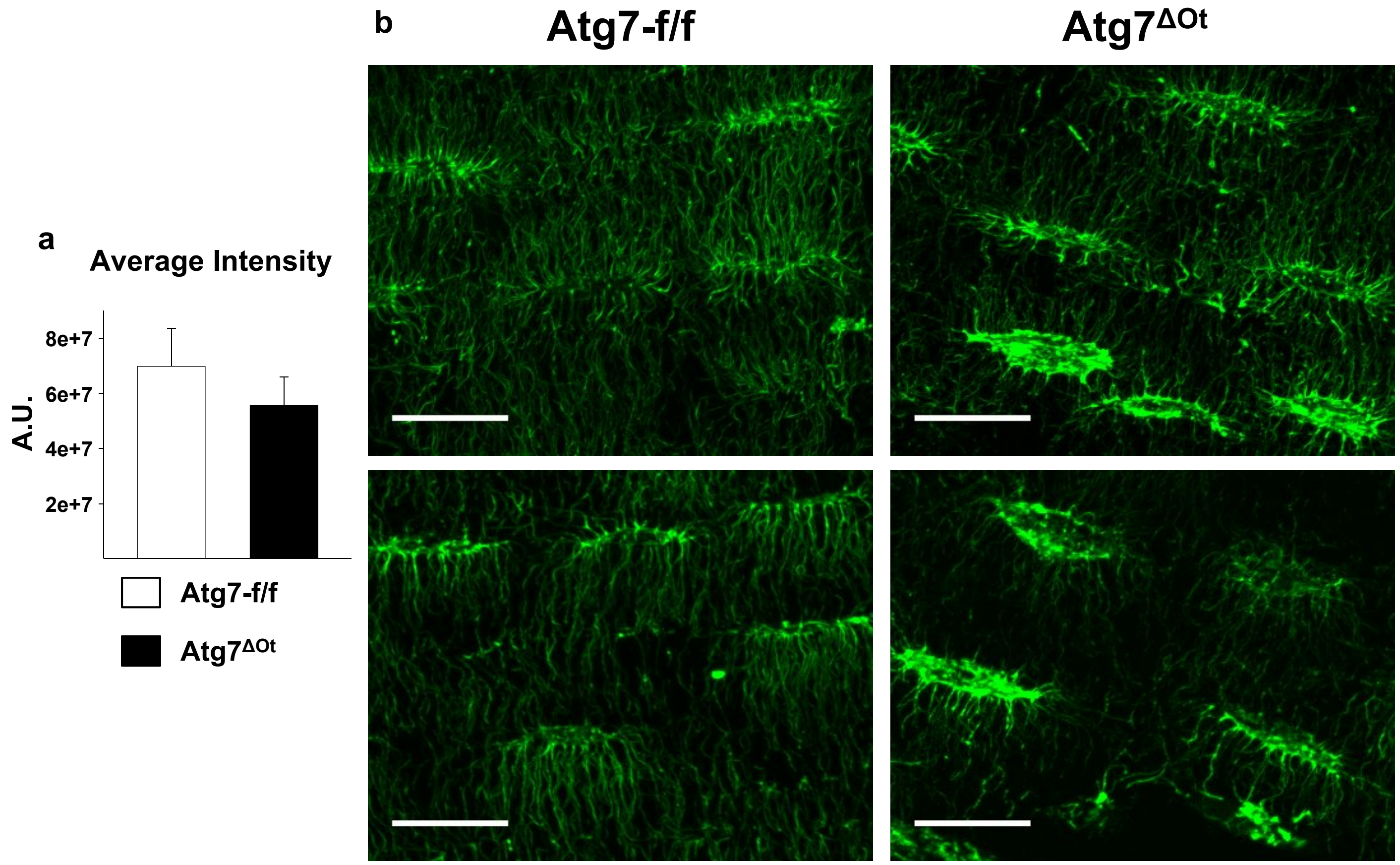

Figure S8. Loss of autophagy in osteocytes does not disrupt the osteocyte network. (**a**) Average intensity of osteocyte projections in tibial cortical bone sections from Atg7-f/f (n = 3) and Atg7Δ^{Ot} (n = 5) mice stained with phallodin-Alexa488. (**b**) Representative images of sections used to obtain values shown in **a**. Each image is of a bone section from different mice and size bar = 20 μm. All measurements were performed in 6-month-old female littermates. Values are the mean ± sd.

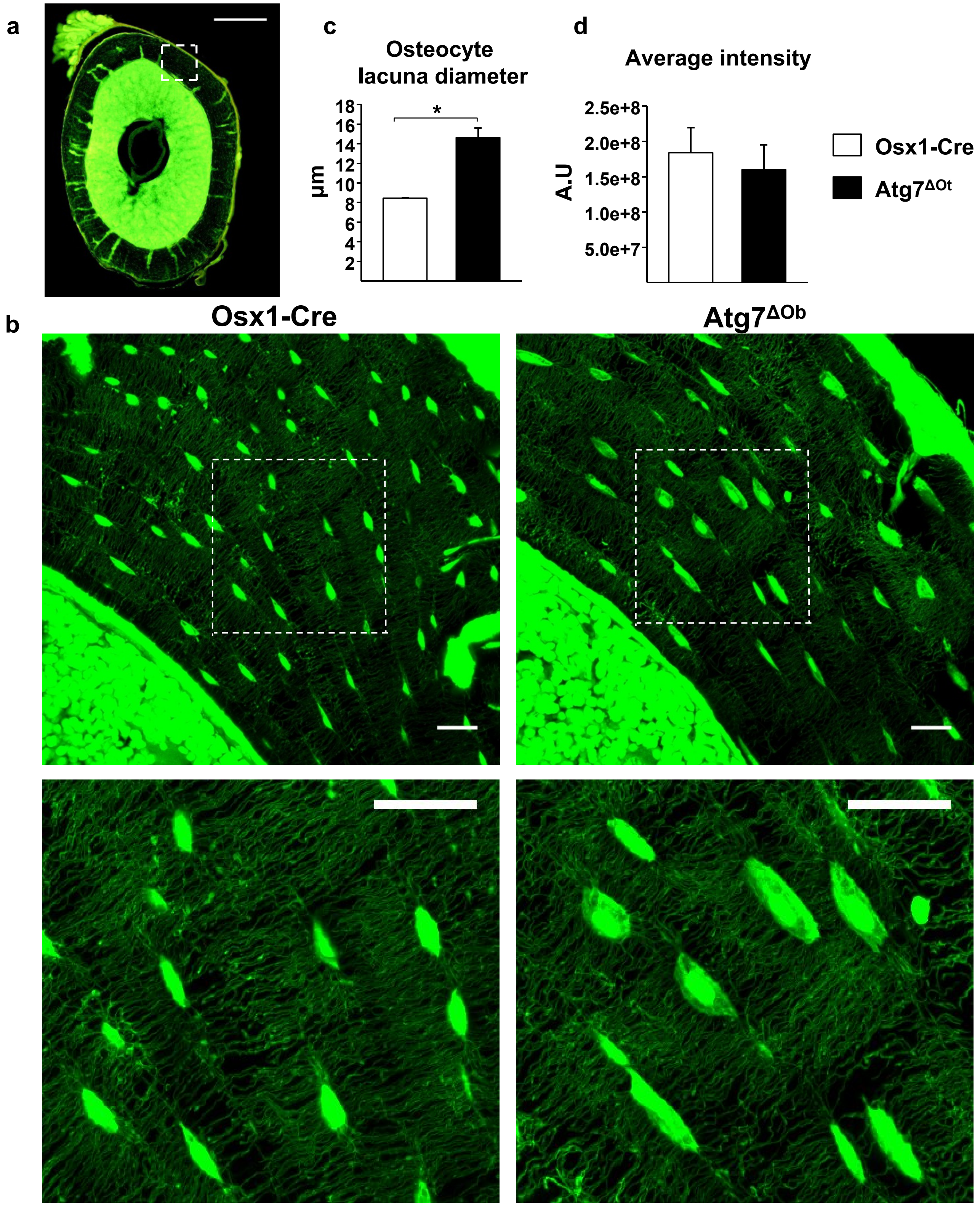

Figure S9. Loss of autophagy in osteoblasts does not alter the canalicular network. (**a**) Low power image of a femoral cross section stained with FITC. White dotted box outlines a representative region of interest showed in **b**. Size bar = 200 μm. (**b**) Representative images of cortical femoral sections stained with FITC used for osteocyte canalicular measurements. Size bar = 20 μm. (**c-d**) Osteocyte lacuna diameter and average intensity of osteocyte canalicular network in femoral cross sections from Osx1-Cre (n = 3) and Atg7$^{\Delta Ot}$ (n = 4) mice stained with FITC from sections described in **b**. All measurements were performed in 6-month-old female littermates. Values are the mean ± sd. *P < 0.05 by Nested analysis.

Table S1

Rate of spontaneous fractures		
	**Atg7$^{\Delta Ob}$**	**mCAT;Atg7$^{\Delta Ob}$**
Animals	17	10
Animals with tibia fractures	12	5
%	70	50
